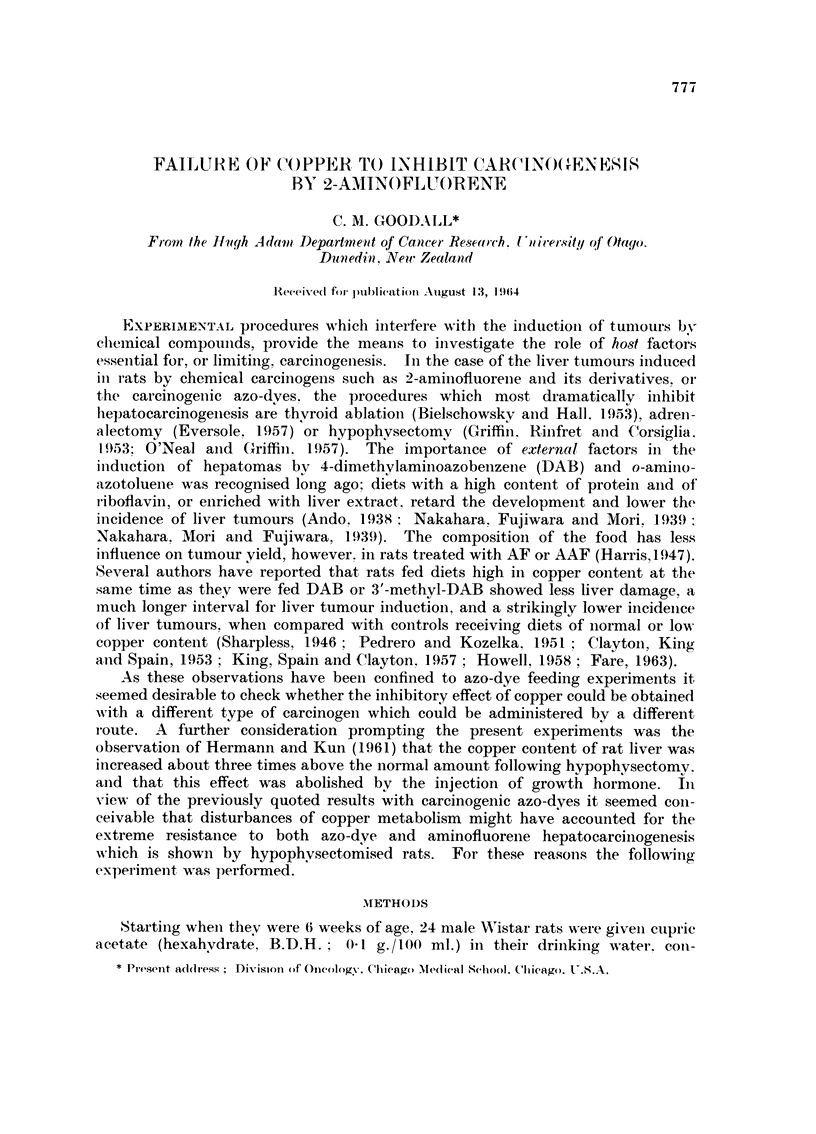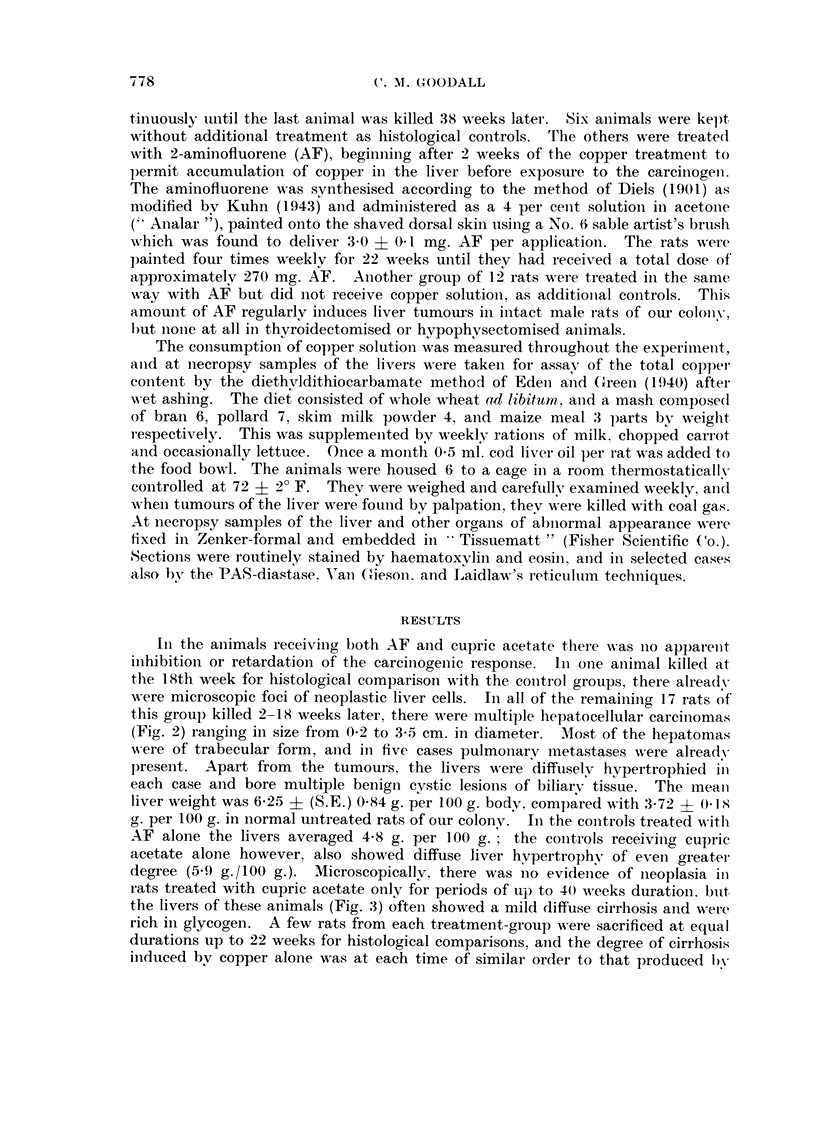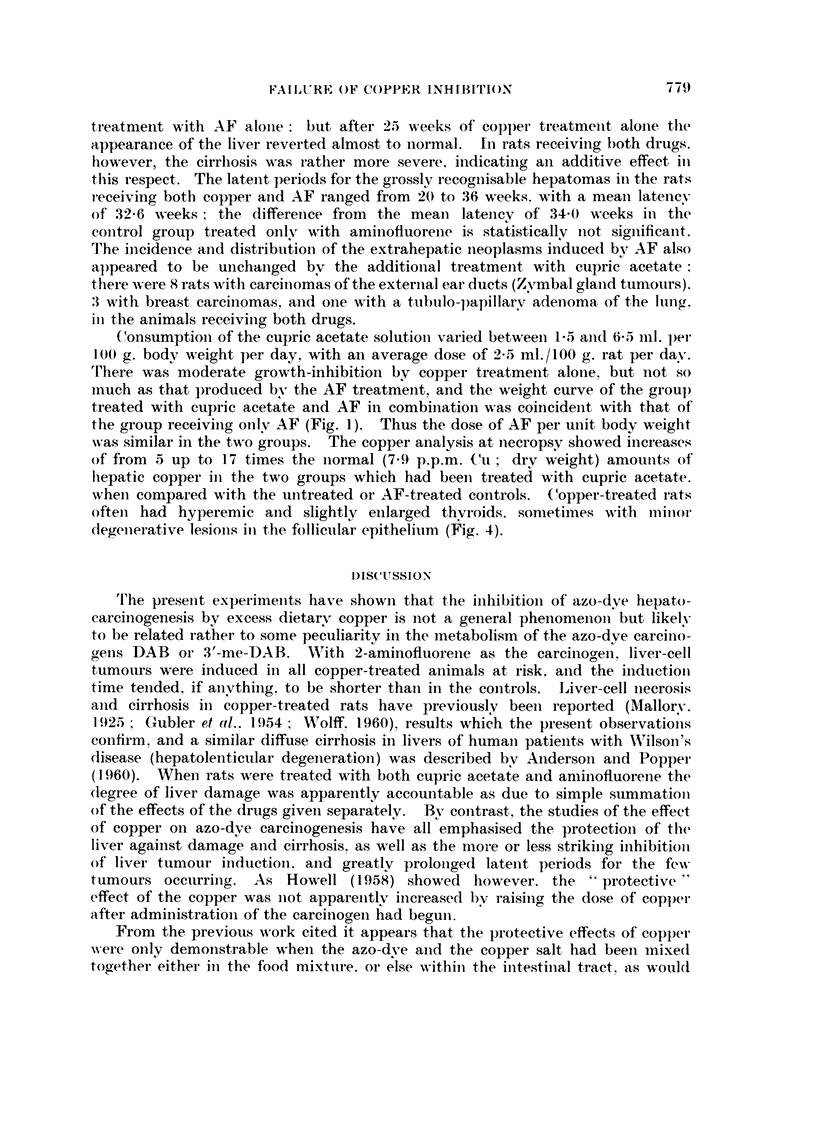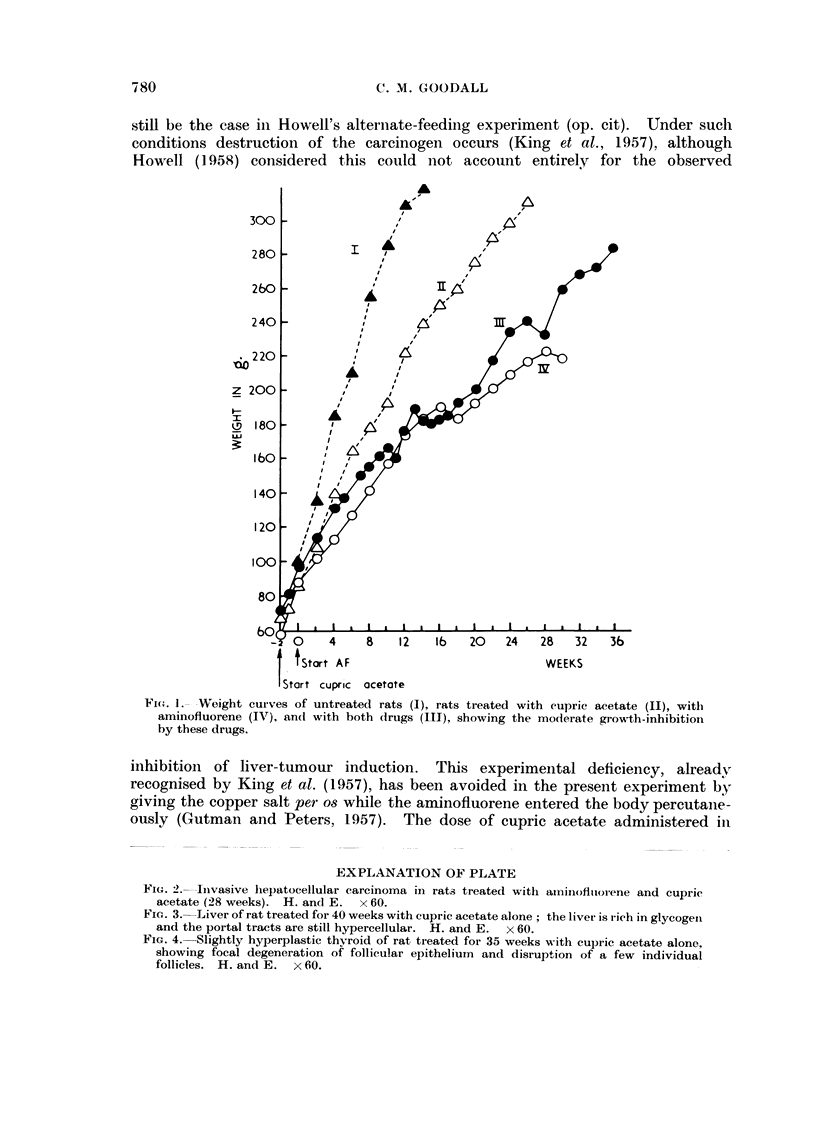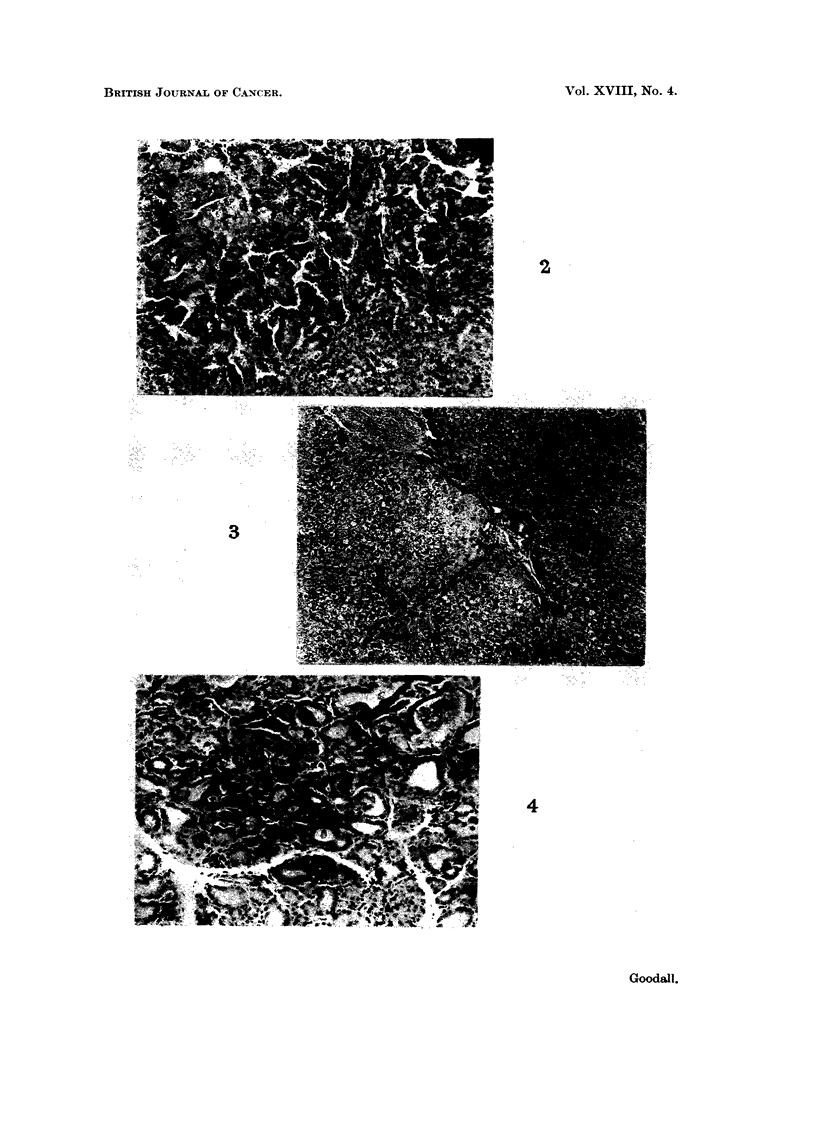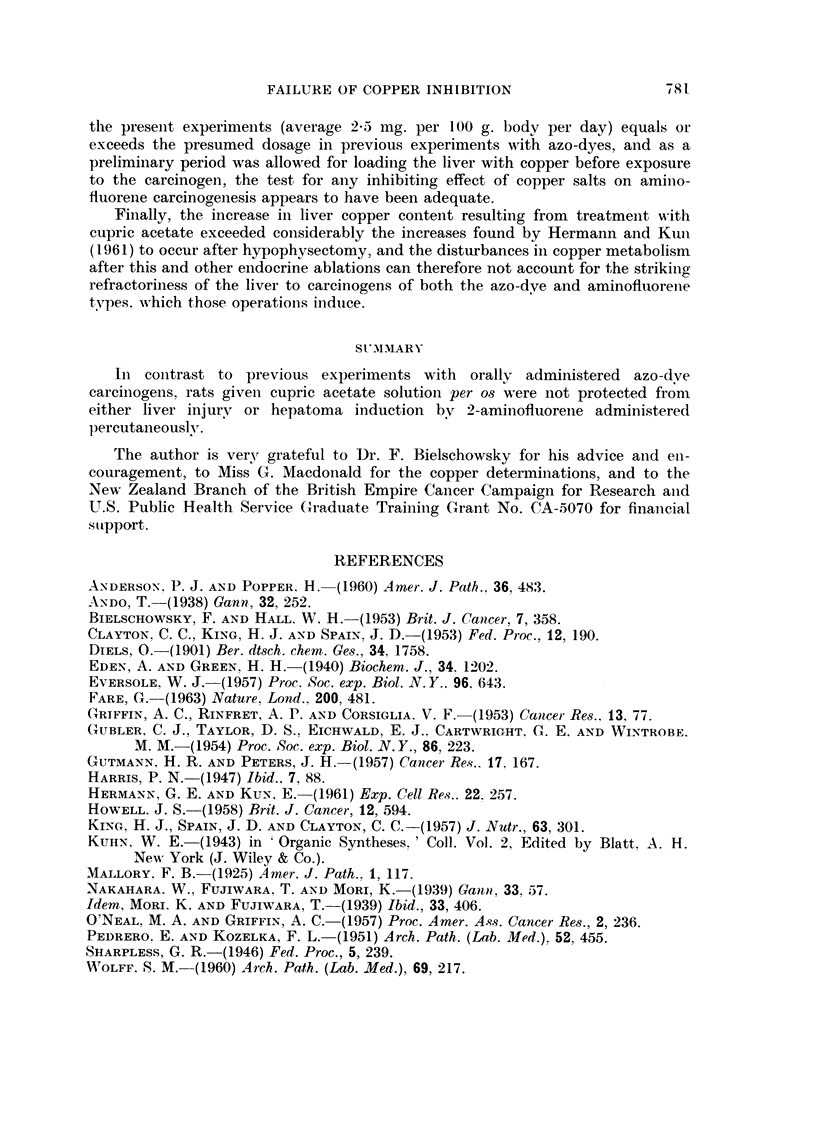# Failure of Copper to Inhibit Carcinogenesis by 2-Aminofluorene

**DOI:** 10.1038/bjc.1964.90

**Published:** 1964-12

**Authors:** C. M. Goodall

## Abstract

**Images:**


### FAILURE OF COPPER TO INHIBIT CARCINOGENESIS BY 2-AMINOFLUORENE

C. M. GOODALL*

*From the Hugh Adam Department of Cancer Research, University of Otago, Dunedin, New Zealand*

Received for publication August 13, 1964

EXPERIMENTAL procedures which interfere with the induction of tumours by chemical compounds, provide the means to investigate the role of *host* factors essential for, or limiting, carcinogenesis. In the case of the liver tumours induced in rats by chemical carcinogens such as 2-aminofluorene and its derivatives, or the carcinogenic azo-dyes, the procedures which most dramatically inhibit hepatocarcinogenesis are thyroid ablation (Bielschowsky and Hall, 1953), adrenalectomy (Eversole, 1957) or hypophysectomy (Griffin, Rinfret and Corsiglia, 1953; O'Neal and Griffin, 1957). The importance of *external* factors in the induction of hepatomas by 4-dimethylaminoazobenzene (DAB) and *o*-aminoazotoluene was recognised long ago; diets with a high content of protein and of riboflavin, or enriched with liver extract, retard the development and lower the incidence of liver tumours (Ando, 1938 ; Nakahara, Fujiwara and Mori, 1939 ; Nakahara, Mori and Fujiwara, 1939). The composition of the food has less influence on tumour yield, however, in rats treated with AF or AAF (Harris,1947). Several authors have reported that rats fed diets high in copper content at the same time as they were fed DAB or 3′-methyl-DAB showed less liver damage, a much longer interval for liver tumour induction, and a strikingly lower incidence of liver tumours, when compared with controls receiving diets of normal or low copper content (Sharpless, 1946 ; Pedrero and Kozelka, 1951 ; Clayton, King and Spain, 1953 ; King, Spain and Clayton, 1957 ; Howell, 1958 ; Fare, 1963).

As these observations have been confined to azo-dye feeding experiments it seemed desirable to check whether the inhibitory effect of copper could be obtained with a different type of carcinogen which could be administered by a different route. A further consideration prompting the present experiments was the observation of Hermann and Kun (1961) that the copper content of rat liver was increased about three times above the normal amount following hypophysectomy, and that this effect was abolished by the injection of growth hormone. In view of the previously quoted results with carcinogenic azo-dyes it seemed conceivable that disturbances of copper metabolism might have accounted for the extreme resistance to both azo-dye and aminofluorene hepatocarcinogenesis which is shown by hypophysectomised rats. For these reasons the following experiment was performed.

### METHODS

Starting when they were 6 weeks of age, 24 male Wistar rats were given cupric acetate (hexahydrate, B.D.H. ; 0·1 g./100 ml.) in their drinking water, con-

* Present address ; Division of Oncology, Chicago Medical School, Chicago, U.S.A.

tinuously until the last animal was killed 38 weeks later. Six animals were kept without additional treatment as histological controls. The others were treated with 2-aminofluorene (AF), beginning after 2 weeks of the copper treatment to permit accumulation of copper in the liver before exposure to the carcinogen. The aminofluorene was synthesised according to the method of Diels (1901) as modified by Kuhn (1943) and administered as a 4 per cent solution in acetone (" Analar "), painted onto the shaved dorsal skin using a No. 6 sable artist's brush which was found to deliver $3{\cdot}0 \pm 0{\cdot}1$ mg. AF per application. The rats were painted four times weekly for 22 weeks until they had received a total dose of approximately 270 mg. AF. Another group of 12 rats were treated in the same way with AF but did not receive copper solution, as additional controls. This amount of AF regularly induces liver tumours in intact male rats of our colony, but none at all in thyroidectomised or hypophysectomised animals.

The consumption of copper solution was measured throughout the experiment, and at necropsy samples of the livers were taken for assay of the total copper content by the diethyldithiocarbamate method of Eden and Green (1940) after wet ashing. The diet consisted of whole wheat *ad libitum*, and a mash composed of bran 6, pollard 7, skim milk powder 4, and maize meal 3 parts by weight respectively. This was supplemented by weekly rations of milk, chopped carrot and occasionally lettuce. Once a month 0·5 ml. cod liver oil per rat was added to the food bowl. The animals were housed 6 to a cage in a room thermostatically controlled at $72 \pm 2°$ F. They were weighed and carefully examined weekly, and when tumours of the liver were found by palpation, they were killed with coal gas. At necropsy samples of the liver and other organs of abnormal appearance were fixed in Zenker-formal and embedded in " Tissuematt " (Fisher Scientific Co.). Sections were routinely stained by haematoxylin and eosin, and in selected cases also by the PAS-diastase, Van Gieson, and Laidlaw's reticulum techniques.

### RESULTS

In the animals receiving both AF and cupric acetate there was no apparent inhibition or retardation of the carcinogenic response. In one animal killed at the 18th week for histological comparison with the control groups, there already were microscopic foci of neoplastic liver cells. In all of the remaining 17 rats of this group killed 2–18 weeks later, there were multiple hepatocellular carcinomas (Fig. 2) ranging in size from 0·2 to 3·5 cm. in diameter. Most of the hepatomas were of trabecular form, and in five cases pulmonary metastases were already present. Apart from the tumours, the livers were diffusely hypertrophied in each case and bore multiple benign cystic lesions of biliary tissue. The mean liver weight was $6{\cdot}25 \pm$ (S.E.) 0·84 g. per 100 g. body, compared with $3{\cdot}72 \pm 0{\cdot}18$ g. per 100 g. in normal untreated rats of our colony. In the controls treated with AF alone the livers averaged 4·8 g. per 100 g. ; the controls receiving cupric acetate alone however, also showed diffuse liver hypertrophy of even greater degree (5·9 g./100 g.). Microscopically, there was no evidence of neoplasia in rats treated with cupric acetate only for periods of up to 40 weeks duration, but the livers of these animals (Fig. 3) often showed a mild diffuse cirrhosis and were rich in glycogen. A few rats from each treatment-group were sacrificed at equal durations up to 22 weeks for histological comparisons, and the degree of cirrhosis induced by copper alone was at each time of similar order to that produced by treatment with AF alone ; but after 25 weeks of copper treatment alone the appearance of the liver reverted almost to normal. In rats receiving both drugs, however, the cirrhosis was rather more severe, indicating an additive effect in this respect. The latent periods for the grossly recognisable hepatomas in the rats receiving both copper and AF ranged from 20 to 36 weeks, with a mean latency of 32·6 weeks ; the difference from the mean latency of 34·0 weeks in the control group treated only with aminofluorene is statistically not significant. The incidence and distribution of the extrahepatic neoplasms induced by AF also appeared to be unchanged by the additional treatment with cupric acetate : there were 8 rats with carcinomas of the external ear ducts (Zymbal gland tumours), 3 with breast carcinomas, and one with a tubulo-papillary adenoma of the lung, in the animals receiving both drugs.

Consumption of the cupric acetate solution varied between 1·5 and 6·5 ml. per 100 g. body weight per day, with an average dose of 2·5 ml./100 g. rat per day. There was moderate growth-inhibition by copper treatment alone, but not so much as that produced by the AF treatment, and the weight curve of the group treated with cupric acetate and AF in combination was coincident with that of the group receiving only AF (Fig. 1). Thus the dose of AF per unit body weight was similar in the two groups. The copper analysis at necropsy showed increases of from 5 up to 17 times the normal (7·9 p.p.m. Cu ; dry weight) amounts of hepatic copper in the two groups which had been treated with cupric acetate, when compared with the untreated or AF-treated controls. Copper-treated rats often had hyperemic and slightly enlarged thyroids, sometimes with minor degenerative lesions in the follicular epithelium (Fig. 4).

### DISCUSSION

The present experiments have shown that the inhibition of azo-dye hepatocarcinogenesis by excess dietary copper is not a general phenomenon but likely to be related rather to some peculiarity in the metabolism of the azo-dye carcinogens DAB or 3′-me-DAB. With 2-aminofluorene as the carcinogen, liver-cell tumours were induced in all copper-treated animals at risk, and the induction time tended, if anything, to be shorter than in the controls. Liver-cell necrosis and cirrhosis in copper-treated rats have previously been reported (Mallory, 1925 ; Gubler *et al.*, 1954 ; Wolff, 1960), results which the present observations confirm, and a similar diffuse cirrhosis in livers of human patients with Wilson's disease (hepatolenticular degeneration) was described by Anderson and Popper (1960). When rats were treated with both cupric acetate and aminofluorene the degree of liver damage was apparently accountable as due to simple summation of the effects of the drugs given separately. By contrast, the studies of the effect of copper on azo-dye carcinogenesis have all emphasised the protection of the liver against damage and cirrhosis, as well as the more or less striking inhibition of liver tumour induction, and greatly prolonged latent periods for the few tumours occurring. As Howell (1958) showed however, the " protective " effect of the copper was not apparently increased by raising the dose of copper after administration of the carcinogen had begun.

From the previous work cited it appears that the protective effects of copper were only demonstrable when the azo-dye and the copper salt had been mixed together either in the food mixture, or else within the intestinal tract, as would still be the case in Howell's alternate-feeding experiment (op. cit). Under such conditions destruction of the carcinogen occurs (King *et al.*, 1957), although Howell (1958) considered this could not account entirely for the observed

FIG. 1.—Weight curves of untreated rats (I), rats treated with cupric acetate (II), with aminofluorene (IV), and with both drugs (III), showing the moderate growth-inhibition by these drugs.

inhibition of liver-tumour induction. This experimental deficiency, already recognised by King *et al.* (1957), has been avoided in the present experiment by giving the copper salt *per os* while the aminofluorene entered the body percutaneously (Gutman and Peters, 1957). The dose of cupric acetate administered in

EXPLANATION OF PLATE

FIG. 2.—Invasive hepatocellular carcinoma in rats treated with aminofluorene and cupric acetate (28 weeks). H. and E. ×60.

FIG. 3.—Liver of rat treated for 40 weeks with cupric acetate alone ; the liver is rich in glycogen and the portal tracts are still hypercellular. H. and E. ×60.

FIG. 4.—Slightly hyperplastic thyroid of rat treated for 35 weeks with cupric acetate alone, showing focal degeneration of follicular epithelium and disruption of a few individual follicles. H. and E. ×60.

2

3

4

Goodall.

the present experiments (average 2·5 mg. per 100 g. body per day) equals or exceeds the presumed dosage in previous experiments with azo-dyes, and as a preliminary period was allowed for loading the liver with copper before exposure to the carcinogen, the test for any inhibiting effect of copper salts on amino-fluorene carcinogenesis appears to have been adequate.

Finally, the increase in liver copper content resulting from treatment with cupric acetate exceeded considerably the increases found by Hermann and Kun (1961) to occur after hypophysectomy, and the disturbances in copper metabolism after this and other endocrine ablations can therefore not account for the striking refractoriness of the liver to carcinogens of both the azo-dye and aminofluorene types, which those operations induce.

### SUMMARY

In contrast to previous experiments with orally administered azo-dye carcinogens, rats given cupric acetate solution *per os* were not protected from either liver injury or hepatoma induction by 2-aminofluorene administered percutaneously.

The author is very grateful to Dr. F. Bielschowsky for his advice and encouragement, to Miss G. Macdonald for the copper determinations, and to the New Zealand Branch of the British Empire Cancer Campaign for Research and U.S. Public Health Service Graduate Training Grant No. CA-5070 for financial support.

### REFERENCES

Anderson, P. J. and Popper, H.—(1960) *Amer. J. Path.*, **36**, 483.
Ando, T.—(1938) *Gann*, **32**, 252.
Bielschowsky, F. and Hall, W. H.—(1953) *Brit. J. Cancer*, **7**, 358.
Clayton, C. C., King, H. J. and Spain, J. D.—(1953) *Fed. Proc.*, **12**, 190.
Diels, O.—(1901) *Ber. dtsch. chem. Ges.*, **34**, 1758.
Eden, A. and Green, H. H.—(1940) *Biochem. J.*, **34**, 1202.
Eversole, W. J.—(1957) *Proc. Soc. exp. Biol. N.Y.*, **96**, 643.
Fare, G.—(1963) *Nature, Lond.*, **200**, 481.
Griffin, A. C., Rinfret, A. P. and Corsiglia, V. F.—(1953) *Cancer Res.*, **13**, 77.
Gubler, C. J., Taylor, D. S., Eichwald, E. J., Cartwright, G. E. and Wintrobe, M. M.—(1954) *Proc. Soc. exp. Biol. N.Y.*, **86**, 223.
Gutmann, H. R. and Peters, J. H.—(1957) *Cancer Res.*, **17**, 167.
Harris, P. N.—(1947) *Ibid.*, **7**, 88.
Hermann, G. E. and Kun, E.—(1961) *Exp. Cell Res.*, **22**, 257.
Howell, J. S.—(1958) *Brit. J. Cancer*, **12**, 594.
King, H. J., Spain, J. D. and Clayton, C. C.—(1957) *J. Nutr.*, **63**, 301.
Kuhn, W. E.—(1943) in 'Organic Syntheses,' Coll. Vol. 2, Edited by Blatt, A. H. New York (J. Wiley & Co.).
Mallory, F. B.—(1925) *Amer. J. Path.*, **1**, 117.
Nakahara, W., Fujiwara, T. and Mori, K.—(1939) *Gann*, **33**, 57.
*Idem*, Mori, K. and Fujiwara, T.—(1939) *Ibid.*, **33**, 406.
O'Neal, M. A. and Griffin, A. C.—(1957) *Proc. Amer. Ass. Cancer Res.*, **2**, 236.
Pedrero, E. and Kozelka, F. L.—(1951) *Arch. Path.* (*Lab. Med.*), **52**, 455.
Sharpless, G. R.—(1946) *Fed. Proc.*, **5**, 239.
Wolff, S. M.—(1960) *Arch. Path.* (*Lab. Med.*), **69**, 217.